# Development and Validation of Claims-based Algorithms for Identifying Hospitalized Patients With COVID-19 and Their Severity in 2020 and 2021

**DOI:** 10.2188/jea.JE20230285

**Published:** 2024-10-05

**Authors:** Chieko Ishiguro, Wataru Mimura, Junko Terada, Nobuaki Matsunaga, Hironori Ishiwari, Hiroyuki Hoshimoto, Kengo Miyo, Norio Ohmagari

**Affiliations:** 1 Center for Clinical Sciences, National Center for Global Health and Medicine, Tokyo, Japan; 2 Center for Respiratory Medicine, National Center for Global Health and Medicine Hospital, Tokyo, Japan; 3 AMR Clinical Reference Center, National Center for Global Health and Medicine, Tokyo, Japan; 4 Center for Medical Informatics Intelligence, National Center for Global Health and Medicine, Tokyo, Japan; 5 Disease Control and Prevention Center, National Center for Global Health and Medicine, Tokyo, Japan

**Keywords:** COVID-19, validation, insurance claims, algorithm, Japan

## Abstract

**Background:**

This study aimed to develop and validate claims-based algorithms for identifying hospitalized patients with coronavirus disease 2019 (COVID-19) and disease severity.

**Methods:**

We used claims data including all patients at the National Center for Global and Medicine Hospital between January 1, 2020, and December 31, 2021. The claims-based algorithms for three statuses with COVID-19 (hospitalizations, moderate or higher status, and severe status) were developed using diagnosis codes (International Classification of Diseases, 10^th^ revision code: U07.1, B34.2) and relevant medical procedure code. True cases were determined using the COVID-19 inpatient registry and electronic health records. Sensitivity, specificity, positive predictive value (PPV), and negative predictive value (NPV) were calculated for each algorithm at 6-month intervals.

**Results:**

Of the 75,711 total patients, the number of true cases was 1,192 for hospitalizations, 622 for moderate or higher status, and 55 for severe status. The diagnosis code-only algorithm for hospitalization had sensitivities 90.4% to 94.9% and PPVs 9.3% to 19.4%. Among the algorithms consisting of both diagnosis codes and procedure codes, high sensitivity and PPV were observed during the following periods: 93.9% and 97.1% for hospitalization (January–June 2021), 90.4% and 87.5% for moderate or higher status (July–December 2021), and 92.3% and 85.7% for severe status (July–December 2020), respectively. Almost all algorithms had specificities and NPVs of approximately 99%.

**Conclusion:**

The diagnosis code-only algorithm for COVID-19 hospitalization showed low validity throughout the study period. The algorithms for hospitalizations, moderate or higher status, and severe status with COVID-19, consisting of both diagnosis codes and procedure codes, showed high validity in some periods.

## INTRODUCTION

Since the outbreak of coronavirus disease 2019 (COVID-19), caused by the severe acute respiratory syndrome coronavirus 2 (SARS-CoV-2), cases of COVID-19 have been increasing in Japan, with over 30 million infected people and over 70,000 deaths reported between January 14, 2020, and March 14, 2023.^1^ Under these circumstances, timely and accurate data on COVID-19 are needed worldwide for policy making or medical procedures. Health insurance claims data are a commonly used data source for real-world evidence.^2^ However, they usually lack the laboratory results required for diagnosing COVID-19.^3^ Therefore, claims-based algorithms with sufficient validity are required to identify patients with COVID-19 as a study cohort, or to identify COVID-19 occurrence as a clinical outcome in epidemiological research using claims data.

Several validation studies have been conducted in different countries to demonstrate the validity of claims-based algorithms for identifying patients with COVID-19 from claims data sources.^4^^–^^6^ In the United States, studies have shown that the algorithm using diagnosis codes (International Classification of Diseases, 10^th^ revision [ICD-10]: U07.1) to identify COVID-19 hospitalization from claims data had a high positive predictive value (PPV) of 84.7% and a sensitivity of 94.9% in the Sentinel System^4^ and a high PPV of 93.8% in the database of the Department of Veterans of Affairs.^5^ However, to our knowledge, no study has validated algorithms identifying COVID-19 in Japan. The validity of claim-based algorithms varies depending on each country’s medical environment and insurance system; as the validity of ICD-10 coded diagnosis information in Japanese claims data is low, it is recommended to combine diagnosis information with medical procedures when creating a claims-based algorithm.^7^^,^^8^

Additionally, most previous validation studies focused on the algorithm for identifying hospitalization for COVID-19, and not many investigated the disease severity in detail. Generally, hospitalization has been regarded as a proxy for severity in epidemiological studies; however, the criteria for hospitalization of COVID-19 patients have changed over time depending on the epidemic waves and the emergence of new treatments. Thus, the use of hospitalization as a definition of study population or outcome in an epidemiological study might not be recommended for certain research purposes owing to the uncertainty from a clinical perspective.^9^ Therefore, a claims-based algorithm for identifying COVID-19 status with severity is essential.

This study aimed to develop and validate algorithms for identifying not only COVID-19 hospitalized patients but also those with moderate or severe status to support future research based on claims data during 2020–2021.

## METHODS

### Study setting and population

This cross-sectional study was conducted using claims data from the National Center for Global Health and Medicine (NCGM) Hospital in Tokyo, Japan. NCGM, a large 749-bed general hospital with 43 departments, is one of the *Designated Medical Institutions for Infectious Disease* by the national government.^10^^,^^11^ All outpatients and inpatients who visited NCGM and received at least one service covered by public health insurance systems between January 1, 2020, and December 30, 2021, were included in the study population.

### Data source

We used the claims data from NCGM Hospital, including data of patients covered by three types of public health insurance systems: the National Health Insurance System (NHI), Medical Care System for the elderly aged 75 years and over, and Employees’ Health Insurance system. The data format for claims data, the “*RECEIPTC*” with a “.UKE” filename extension, is the same across all health insurance systems in Japan, including four types: *Medical claims* data (fee-for-service for inpatients and outpatients), *Diagnosis Procedure Combination (DPC) claims* data (comprehensive payment system for inpatients), *Dental claims* data, and *Dispensing claims* data.^12^ We used the nationwide COVID-19 inpatient registry (COVIREGI-JP), which was established and maintained by the Center for Disease Control and Prevention in NCGM, as the reference standard data for validation.^13^ COVIREGI-JP, which registered all hospitalized patients with COVID-19 in NCGM (inclusion criteria: positive SARS-CoV-2 test and inpatient treatment at a healthcare facility) since January 2020, includes their clinical and treatment information collected using the case report form based on the International Severe Acute Respiratory and Emerging Infection Consortium (ISARIC).^14^ The COVIREGI- JP data about inpatients at NCGM are linked to the NCGM claims database on an individual basis. However, information on participants for clinical trials or specific clinical studies registered in the COVIREGI-JP was not available for research purposes. Therefore, their information was obtained through reviewing their electronic medical records by a medical doctor.

### Health state being validated and reference case

Three patterns of COVID-19 status were targeted to validate: hospitalizations, hospitalizations of moderate or higher status, and hospitalizations of severe status. These three groups were not exclusive and there were duplications. Our reference cases for hospitalizations with COVID-19 comprised all inpatients who tested positive for SARS-CoV-2. This was consistent with the criteria for COVIREGI-JP^®^ registration at this hospital. COVID-19 severity per hospitalization in this study was defined by the procedure for oxygen support treatment recorded in the COVIREGI-JP^®^, based on a previous study.^13^ Our reference cases of “moderate or higher” status comprised those patients who, on at least one occasion, received noninvasive mechanical ventilation, supplemental oxygen (including high-flow oxygen devices), invasive mechanical ventilation (IMV), or extracorporeal membrane oxygenation (ECMO) during hospitalization. Our reference cases of “severe” status comprised those patients who received IMV or ECMO on at least one occasion during hospitalization. These criteria for COVIREG-JP^®^ registration were not changed in this hospital during the study period.

### Algorithms

The identification algorithms consisted of a combination of several factors from claims data, as shown in Table 1. To identify hospitalized patients with COVID-19, we developed four algorithms based on diagnosis codes (ICD-10) and medical procedure codes (the national standardized code for electronic claims data processing systems). Algorithm-1 consisted of diagnosis code U07.1 (COVID-19) or B34.2 (coronavirus infection) excluding suspected diagnosis; Algorithm-2 consisted of Algorqithm-1 and medical procedure codes for additional charges of *Category-II Infectious Diseases*, which were added when the patient was diagnosed with COVID-19, poliomyelitis, tuberculosis, diphtheria, severe acute respiratory syndrome, Middle East respiratory syndrome, or avian influenza (H5N1 or H7N9).^15^ Algorithm-3 consisted of Algorithm-1 and the medical procedure codes for admission, medical supervision, and additional charges related to COVID-19 treatment, which were added when COVID-19 was diagnosed.^16^ Since February 2020, many medical procedure codes for charges related to COVID-19 treatment have been continually issued as tentative codes and then discontinued. We used all medical procedure codes for charges related to COVID-19 treatment that were issued during the study period (eTable 1). Algorithm-4 consisted of Algorithm-1 and medical procedure codes for charges specific to COVID-19 treatment or *Category-II Infectious Diseases*. Algorithms based on the severity of COVID-19 were developed from Algorithms 3 and 4.

**Table 1.** Claims-based algorithms for hospitalization, moderate or higher status, and severe status with COVID-19

Health status	Algorithm	Diagnosis^a^	Medical procedure for admission, medical supervision, or additional charges^b^	Medical procedure for treatment^b^
Hospitalization	#1	U07.1, B34.2		
	#2	U07.1, B34.2	Category-II Infectious Disease	
	#3	U07.1, B34.2	COVID-19-related codes	
	#4	U07.1, B34.2	COVID-19-related codes or Category-II Infectious Disease	

Moderate or higher	#3M	U07.1, B34.2	COVID-19-related codes	NIMV, supplemental oxygen, IMV, or ECMO
	#4M	U07.1, B34.2	COVID-19-related codes or Category-II Infectious Disease	NIMV, supplemental oxygen, IMV, or ECMO

Severe	#3S	U07.1, B34.2	COVID-19-related code	IMV or ECMO
	#4S	U07.1, B34.2	COVID-19-related codes or Category-II Infectious Disease	IMV or ECMO

COVID-19, coronavirus disease 2019; ECMO, extracorporeal membrane oxygenation; IMV, invasive mechanical ventilation; NIMV, noninvasive mechanical ventilation.

Index date of each algorithm was defined as claims-based hospital admission date.

^a^Diagnosis: identified by the International Classification of Diseases, 10th Revision (ICD-10) code.

^b^Medical procedure: identified by the national standardized code for electronic claims data processing system.

For moderate or higher status, Algorisms 3M and 4M were developed by combining treatment medical procedure codes (noninvasive mechanical ventilation, supplemental oxygen, IMV, or ECMO) during hospitalization with Algorithms-3 or 4, respectively. For severe status, Algorithms 3S and 4S were developed by combining IMV or ECMO during hospitalization with Algorithms 3 or 4, respectively. For all algorithms, information on diagnoses and procedures was obtained from *Medical claims* data for inpatients, which included all COVID-19 treatments for inpatients at the study hospital during the study period. The index date was determined as the earliest hospital admission date for each patient qualified by each algorithm that met the algorithm criteria in the claims data.

### Validity measures

**Figure 1.**
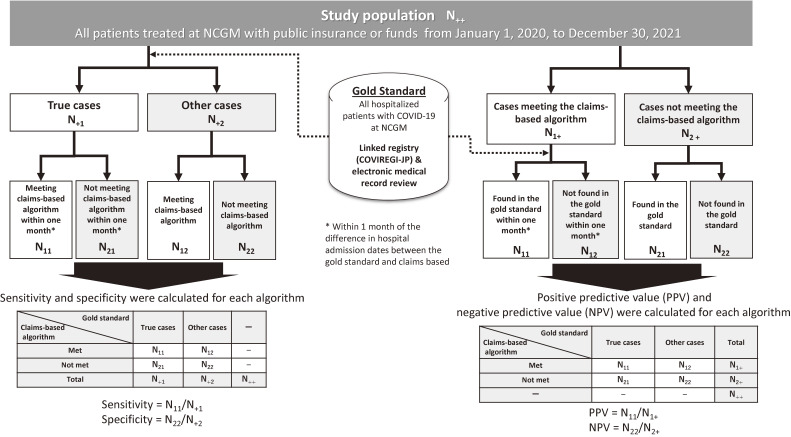
Study flow chart. COVIREGI-JP, COVID-19 inpatient registry; NCGM, National Center for Global and Medicine Hospital; NPV, negative predictive value; PPV, positive predictive value.

The study process for estimating PPV, negative predictive value (NPV), sensitivity, and specificity is shown in Figure 1. For estimating PPV and NPV, we identified patients qualified by each algorithm and their first index dates from claims data. Subsequently we confirmed whether these patients were true COVID-19 inpatients or not by referring to COVIREGI-JP or by reviewing the medical records. For estimating the sensitivity and specificity of the algorithms, we also identified all hospitalized patients with COVID-19 and the earliest hospital admission date from the COVIREGI-JP or medical record review. Subsequently we assessed whether these patients were identified by each algorithm.

### Statistical analysis

For the whole study population and the sub-population of the true hospitalized patients with COVID-19, demographics and disease classifications based on ICD-10 diagnosis codes were tabulated. Information on disease classifications was assessed for the entire study period for the study population or prior to the month of hospital admission for true COVID-19 inpatients. The sensitivity, specificity, PPV, and NPV per each algorithm were calculated over the study period and at 6-month intervals. Exact Clopper–Pearson confidence intervals for all validity indices were calculated. Analysis was conducted using R version 4.1.2 (R Foundation for Statistical Computing, Vienna, Austria).

### Additional analyses

Algorithms 3S’ and 4S’ were developed to identify the time of change to severe status after hospitalization based on Algorithms 3S and 4S by restricting the procedure date of IMV or ECMO to the second day of hospitalization or later. True determination was based on the consistency of the procedure date between the claims data and the registry data. PPVs were subsequently estimated.

### Ethics

The Institutional Review Board at NCGM approved the study protocol (No. NCGM-S-004428-00). Informed consent was applied using the opt-out method because patients were included in this study from claims data, registry, and medical records.

## RESULTS

### Patients

A total of 75,711 patients received publicly insured care at NCGM between January 2020 and December 2021. True hospitalized patients with COVID-19 in NCGM comprised 1,192 individuals identified from the COVIREGI-JP or medical record review across the entire study period. Of these, 622 patients were identified as cases with moderate or higher status, and 55 patients were identified as cases with severe status. Table 2 shows the demographics and disease classifications of true hospitalized patients and the total study population. The diseases that were more common among the hospitalized patients with COVID-19 (registry-based definition) than in the total study population were: “A00-B99 Certain infectious and parasitic diseases,” “D50-89 Diseases of the blood and blood-forming organs and certain diseases affecting the immune mechanism,” “E00-E90 Endocrine, nutritional and metabolic diseases,” “J00-J99 Diseases of the respiratory system,” and “N00-N99 Diseases of the genitourinary system.”

**Table 2.** Demographics and disease characteristics of the study population and true hospitalized patients with COVID-19

	STUDY POPULATION *N* = 75,711	HOSPITALIZED PATIENTS WITH COVID-19 *N* = 1,192
**Sex**		
Male	37,238 (49.2%)	770 (64.5%)
**Age, years**		
Median (IQR)	55 (34–73)	53 (39–68)
<16	6,471 (8.5%)	17 (1.4%)
16–64	40,546 (53.6%)	808 (67.7%)
≥65	28,694 (37.9%)	368 (30.8%)
**Disease characteristics** ^a^		
A00-B99 Certain infectious and parasitic diseases	27,719 (36.6%)	838 (70.2%)
C00-D48 Neoplasms	27,319 (36.1%)	180 (15.1%)
D50-D89 Diseases of the blood and blood-forming organs and certain disorders ​ involving the immune mechanism	27,135 (35.8%)	908 (76.1%)
E00-E90 Endocrine, nutritional and metabolic diseases	41,247 (54.5%)	983 (82.4%)
F00-F99 Mental and behavioral disorders	12,175 (16.1%)	219 (18.4%)
G00-G99 Diseases of the nervous system	21,236 (28.0%)	434 (36.4%)
H00-H59 Diseases of the eye and adnexa	14,057 (18.6%)	137 (11.5%)
H60-H95 Diseases of the ear and mastoid process	4,742 (6.3%)	25 (2.1%)
I00-I99 Diseases of the circulatory system	42,763 (56.5%)	760 (63.7%)
J00-J99 Diseases of the respiratory system	29,545 (39.0%)	754 (63.2%)
K00-K93 Diseases of the digestive system	40,473 (53.5%)	793 (66.5%)
L00-L99 Diseases of the skin and subcutaneous tissue	18,826 (24.9%)	294 (24.6%)
M00-M99 Diseases of the musculoskeletal system and connective tissue	25,921 (34.2%)	517 (43.3%)
N00-N99 Diseases of the genitourinary system	24,311 (32.1%)	969 (81.2%)
O00-O99 Pregnancy, childbirth and the puerperium	2,559 (3.4%)	15 (1.3%)
P00-P96 Certain conditions originating in the perinatal period	1,081 (1.4%)	3 (0.3%)
Q00-Q99 Congenital malformations, deformations and chromosomal abnormalities	2,260 (3.0%)	9 (0.8%)
R00-R99 Symptoms, signs and abnormal clinical and laboratory findings, ​ not elsewhere classified	36,447 (48.1%)	392 (32.9%)
S00-T98 Injury, poisoning and certain other consequences of external causes	17,686 (23.4%)	127 (10.6%)
V00-Y98 External causes of morbidity and mortality	1,767 (2.3%)	26 (2.2%)
Z00-Z99 Factors influencing health status and contact with health services	6,837 (9.0%)	67 (5.6%)

COVID-19, coronavirus disease 2019; IQR, interquartile range.

^a^Disease characteristics were assessed by prior to the month of COVID-19 admission for true cases (COVID-19 inpatients) or by the entire study period for total population.

### Validity of claims-based algorithms

**Figure 2.**
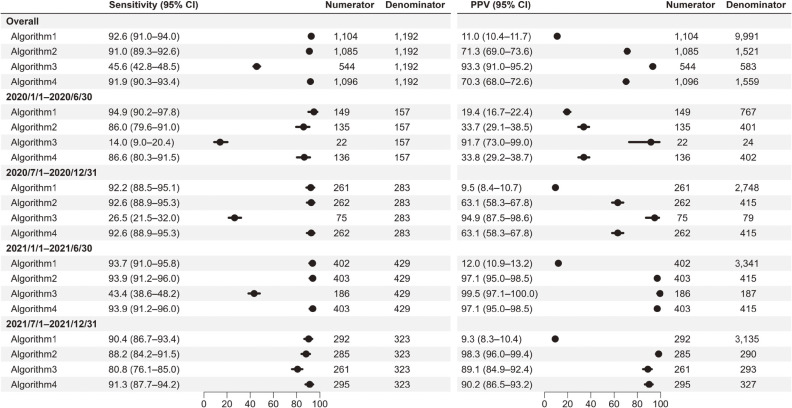
Sensitivity and positive predictive value of each algorithm for hospitalization with COVID-19. CI, confidence interval; PPV, positive predictive value.

The validity of the claims-based algorithms for hospitalization is shown in Figure 2 (sensitivity and PPV) and eFigure 1 (specificity and NPV). In the analysis over the study period, the sensitivity and PPV of the diagnosis code-only algorithm for hospitalization was 92.6% and 11.0%, respectively. The algorithm comprising diagnosis and procedures improved the PPV (Algorithm-4: sensitivity 91.9% and PPV 70.3%). In the 6-month interval analyses, the sensitivity of Algorithm-1, -2, and -4 was consistently high throughout the study period (86.0–94.9%). The sensitivity of Algorithm-3, which was the lowest in the first half of 2020 (14.0%), gradually increased and was 80.8% in late 2021. The PPV of Algorithm-1 was substantially low (9.3–19.4%) throughout the study period. The PPV of Algorithm-2 and -4 gradually increased during the study period. The PPV of Algorithm-3 was high consistently throughout the study period. All algorithms except Algorithm-1 had specificities and NPVs greater than 99%.

**Figure 3.**
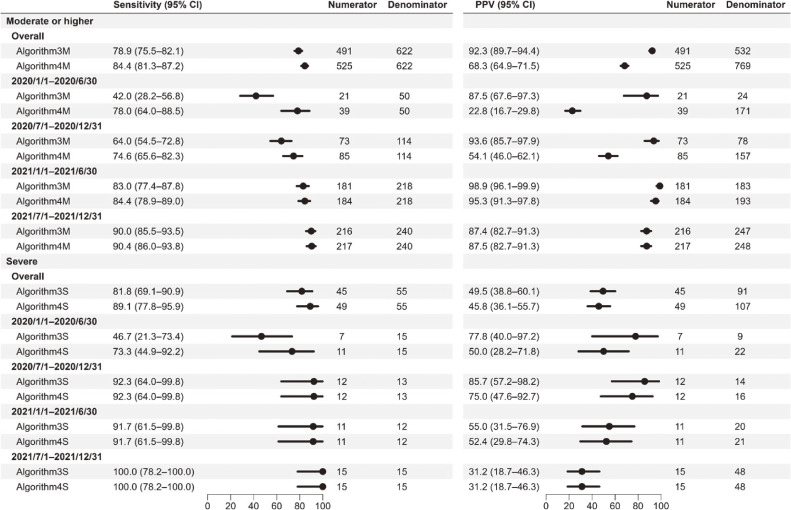
Sensitivity and positive predictive value of each algorithm for moderate or higher and severe status during hospitalization. “Moderate or higher” status includes “Severe” status. CI, confidence interval; PPV, positive predictive value.

The validity of claims-based algorithms for moderate or higher and severe status is shown in Figure 3 (sensitivity and PPV) and eFigure 2 (specificity and NPV). In the analysis over the study period, the highest sensitivity and PPV among the algorithms comprising diagnosis and procedures were 84.4% and 68.3%, respectively, for hospitalization with moderate or higher status, and 89.1% and 45.8%, respectively, for hospitalization with severe status. In the 6-month interval analyses, regarding the sensitivity during the study period, for Algorithm-3M and -3S, the sensitivity was less than 50% in the first half of 2020, which increased gradually, and that for Algorithm-4M and -4S was consistently high. Regarding the PPV during the study period, Algorithm-3M had a consistently high PPV (87.4–98.9%), the PPV of Algorithm-4M gradually increased, that of Algorithm-3S decreased, and that for Algorithm-4S was consistently low. Specificities and NPVs for all algorithms were above 99%. The additional analyses showed that Algorithm-3S’ had a PPV of 31.4% (19.1–45.9%) [N_11_ = 16, N_1+_ = 51], while Algorithm-4S’ had a PPV of 28.8% (18.3–41.3%) [N_11_ = 19, N_1+_ = 66].

## DISCUSSION

To the best of our knowledge, this is the first study to reveal the validity of claims-based algorithms identifying COVID-19 hospitalization in Japan from January 2020 to December 2021. In addition, we developed and validated algorithms for each clinical status (all hospitalization, hospitalization of moderate or higher status, and hospitalization of severe status) of COVID-19 using the registry or medical records. The PPV of the algorithms consisting of only ICD-10 codes (U07.1, B34.2) was extremely low for all statuses; however, the algorithms were improved by combining ICD-10 codes with medical procedure codes for charges specific to COVID-19 treatment.

Among the background factors of the study population, diseases with a high proportion in the true cases (excluding urological diseases) are known risk factors for severe COVID-19.^17^^,^^18^ Urological diseases are not known as risk factors for severe COVID-19, but occurred in a high proportion of patients in the true case group. This is because tubular markers (L-FABP and uβ2MG) were considered useful as early detective biomarkers for COVID-19-associated renal injury^19^ and urinalysis was actively performed in outpatient clinical practice prior to COVID-19 admission at that time.

Regarding the validation of algorithms for hospitalization, Algorithm-1 (ICD-10: U07.1, B34.2 in *Medical claims* data for inpatient) was the broadest algorithm and showed the highest sensitivity; however, the PPV was very low. This might be because these ICD-10 codes were added to conduct laboratory tests for COVID-19 diagnosis, regardless of test results, during hospital admissions to treat other diseases. The sensitivity of Algorithm-2, which combines diagnosis with the codes for an additional fee for *Category-II Infectious Disease*, was also high. Meanwhile, the PPV of Algorithm-2 increased during the study period. Given the existence of additional codes for *Category-II Infectious Disease* prior to the pandemic, these codes may have been assigned to patients suspected of having COVID-19 in the early stages, leading to high sensitivity and low PPVs in 2020. As diagnostic and billing practices became established in 2021, these codes were increasingly allocated to patients with confirmed COVID-19, potentially leading to an increase in PPV. The results were not influenced by the number of patients with *Category II infections* other than COVID-19. In Algorithm-3, the PPV was improved by combining diagnosis with medical procedures (additional fee of COVID-19 treatment); however, there was a significantly low sensitivity in the first half of 2020. This might be because most of these codes were issued in this period; hence, many of them had not been used yet at that time. The sensitivity of Algorithm-4 was higher than that of Algorithms 2 or 3, and the PPV of Algorithm-4 was higher than that of Algorithm-1; however, the PPV in 2020 was low, the same as that of Algorithm-2. Therefore, Algorithm-4 is considered the best choice to identify hospitalized patients with COVID-19 in studies using claims data.

In the validation of algorithms for moderate or higher status, the PPV and sensitivity of Algorithms 3M and 4M improved in 2021, and the same trend was observed in Algorithms 3 and 4 for hospitalization. In contrast, in Algorithms 3S and 4S for severe status, the sensitivity increased but the PPV continued to decrease during the study period. The PPVs of algorithms 3S’ and 4S’ were lower than those of algorithms 3S and 4S, respectively, primarily due to stricter criteria for true cases. Most of the false positives by the algorithms for severe status were patients with non-COVID-19 diseases. This may be because the procedures included only in the moderate or higher status algorithms were more specifically performed on COVID-19 patients than those included in the algorithms for severe status. In addition, the false positives in the algorithms for severe status also included some COVID-19 patients who were identified as receiving IMV in the claims-based algorithm but did not truly receive IMV. This is thought to have been influenced by the Japanese insurance claims rules. Specifically, the cost of a nasal mask for patients with PaO_2_/FIO_2_ less than 300 mm Hg or PaCO_2_ greater than 45 mm Hg, was the same as that of IMV,^20^ and these cases might have been coded as IMV.

In this study, the information about medication for COVID-19 treatment could not be included in claims-based algorithms. In Japan, many medicines for COVID-19 treatments were approved by the Ministry of Health, Labour and Welfare during the study period, such as remdesivir (Veklury^®^) in May 2020, dexamethasone in July 2020, baricitinib (Olumiant^®^) in April 2021, casirivimab/imdevimab (Lonaprev^®^) in July 2021, sotrovimab (Xevudy^®^) in September 2021, and molnupiravir (Lagevrio^®^) in December 2021.^21^ However, most of these medicines were not in the claims data because they were owned by the MHLW and transferred free of charge upon request from medical institutions and pharmacies during the study period.^22^ Once a stable supply is available and general distribution begins, they will be included in the claims data, and we can include medical treatment in the claims-based algorithms and a more valid algorithm could be created.

There are some limitations to this study. First, the external validity of this result is limited because we used claims data derived from one hospital, which does not represent the whole claims data from Japanese healthcare systems. The use of payer-based claims data linked to reference standard data sources would be the best for validation studies; however, hospital-based validation studies are common in Japan^8^^,^^23^^–^^25^ because privacy laws in Japan prohibit the identification of patients directly from those databases. Given the possibility of variations in claims data preparation procedures within our study hospital compared to other hospitals, it is important to acknowledge that the extent of misclassification may differ when applying these algorithms to alternative claims databases. Additionally, it is worth noting that since these algorithms and the reference standard data were developed based on the assessment of illness severity during hospitalization at a single hospital, it is not feasible to track patients' conditions before and after their hospitalization. In addition, as the study hospital is a *Designated Medical Institutions for Infectious Disease*, it is possible that the prevalence of COVID-19 in the study population was higher than that in general hospitals, thereby resulting in a higher PPV. In contrast, sensitivity and specificity are not affected by prevalence and were estimated using a sufficient sample size (study population: >70,000, true cases: >1,000). Second, the results are for a limited period, between January 2020 and December 2021. The COVID-19 pandemic is still ongoing, mutant strains continue, and treatment options and prevalence are likely to continue to change. Therefore, there is a need to continue performing validation for COVID-19 algorithms on a regular basis in the future. Third, our reference cases in this study was based on the fact that a patient was hospitalized for COVID-19 and the most critical procedure observed during that hospitalization. Therefore, further research is needed to develop algorithms for identifying clinical symptoms and changes in severity status during hospitalization. Last, this study covered algorithms for inpatient outcomes only. A further validation study is needed for the COVID-19 outpatient algorithm.

In conclusion, the diagnosis code-only algorithm for COVID-19 hospitalization showed low validity throughout the study period. The algorithms for hospitalizations, moderate or higher status, and severe status with COVID-19, consisting of both diagnosis codes and medical procedure codes, showed high validity in some periods. Our results will support further research using claims data to generate new evidence on COVID-19 in a Japanese setting.

## SUPPLEMENTARY MATERIAL

eTable 1. Code list for medical procedure codes

eFigure 1. Specificities and NPV of each algorithm for hospitalization with COVID-19. CI, confidence interval; COVID-19, coronavirus disease 2019; NPV, negative predictive value.

eFigure 2. Specificities and NPV for each algorithm for moderate or higher and severe status during hospitalization. “Moderate or higher” status includes “Severe” status. CI, confidence interval; NPV, negative predictive value.
