# Supplementary material for: Development and Validation of Claims-based Algorithms for Identifying Hospitalized Patients With COVID-19 and Their Severity in 2020 and 2021
Source: J Epidemiol. 2024 Oct 5;34(10):485–92. doi: 10.2188/jea.JE20230285 (PMC11405369; doi:10.2188/jea.JE20230285)
Supplement: Supplementary file 1 [file je-34-485-s001.pdf]

eTable 1. Code list for medical procedure codes

Category II infectious disease	
Category II infectious disease	190101870, 190127710, 190136210, 190232670, 190237850, 190237550
COVID-19	
COVID-19-related additional charges for acute care management	190221350, 190225850, 190237950, 190232650, 190238050, 190232810, 180065650, 180065850, 114052070, 114053350, 180065750, 180065950, 180065250, 114052270, 114052170,
COVID-19-related additional charges for infants	114052370, 114052570, 114052770, 114053470, 180065370, 180066170,
COVID-19-related additional charges for Category II Infectious Diseases	190232670, 190237850, 190237550,
COVID-19-related additional charges for children	114052470, 114052670, 114052870, 114053570, 180065470, 180066270,
COVID-19-related hospitalization charges for emergency department	190222610, 190222710, 190222810, 190222910, 193522910, 193526010, 193529010, 193549210, 193552210, 193555210, 193523010, 193526110, 193529110, 193549310, 193552310, 193555310, 193522610, 193523110, 193525710, 193528710, 193551910, 193554910, 193522710, 193525810, 193528810, 193549010, 193552010, 193555010, 193522810, 193525910, 193528910, 193549110, 193552110, 193555110, 190221450, 190225950, 190221650, 190226150, 190232910, 190234610, 190221550, 190226050, 193573910, 193574510, 193575110, 193580210, 193580810, 193581410, 193523510, 193526510, 193529510, 193549710, 193552710, 193555710, 193523610, 193526610, 193529610, 193549810, 193552810, 193555810, 193523210, 193526210, 193529210, 193549410, 193552410, 193555410, 193523310, 193526310, 193529310, 193549510, 193552510, 193555510, 193523410, 193526410, 193529410, 193549610, 193552610, 193555610, 190221750, 190226250, 190221950, 190226450, 190233010, 190234710, 190221850, 190226350, 193574010, 193574610, 193575210, 193580310, 193580910, 193581510, 190222050, 190226550, 190222150, 190226650, 190222250, 190226750, 190233110, 190234810, 193524010, 193527010, 193530010, 193550210, 193553210, 193556210, 193524110, 193527110, 193530110, 193550310, 193553310, 193556310, 193523710, 193526710, 193529710, 193549910, 193552910, 193555910, 193523810, 193526810, 193529810, 193550010, 193553010, 193556010, 193523910, 193526910, 193529910, 193550110, 193553110, 193556110, 193574110, 193574710, 193575310, 193580410, 193581010, 193581610, 193524510, 193527510, 193530510, 193550710, 193553710, 193556710, 193524610, 193527610, 193530610, 193550810, 193553810, 193556810, 190228910, 193524210, 193527210, 193530210, 193550410, 193553410, 193556410, 193524310, 193527310, 193530310, 193550510, 193553510, 193556510, 190229010, 190229310, 193524410, 193527410, 193530410, 193550610, 193553610, 193556610, 190229110, 190229410, 190236310, 190236410, 193574210, 193574810, 193575410, 193580510, 193581110, 193581710, 190229210, 190222350, 190226850, 190222450, 190226950, 190222550, 190227050, 190233210, 190234910, 193525010, 193528010, 193531010, 193551210, 193554210, 193557210, 193525110, 193528110, 193531110, 193551310, 193554310, 193557310, 193524710, 193527710, 193530710, 193550910, 193553910, 193556910, 193524810, 193527810, 193530810, 193551010, 193554010, 193557010, 193524910, 193527910, 193530910, 193551110, 193554110, 193557110, 193574310, 193574910, 193575510, 193580610, 193581210, 193581810, 193525510, 193528510, 193531510, 193551710, 193554710, 193557710, 190229810, 190230210, 193525610, 193528610, 193531610, 193551810, 193554810, 193557810, 190229510, 193525210, 193528210, 193531210, 193551410, 193554410, 193557410, 193525310, 193528310, 193531310, 193551510, 193554510, 193557510, 190229610, 190230010, 193525410, 193528410, 193531410, 193551610, 193554610, 193557610, 190229710, 190230110, 190236510, 190236610, 193574410,

	193575010, 193575610, 193580710, 193581310, 193581910, 190229910,
COVID-19-related management charges for growth care unit	193547410, 193547710, 193548010, 193573010, 193573310, 193573610, 193547510, 193547810, 193548110, 193573110, 193573410, 193573710, 193547610, 193547910, 193548210, 193573210, 193573510, 193573810, 193579910, 193580010, 193580110, 193586210, 193586310, 193586410, 190225750, 190228850, 190234510, 190236210,
COVID-19-related management charges for High care unit	160227050, 193539410, 193540210, 193541010, 193565510, 193566110, 193566710, 193539510, 193540310, 193541110, 193565610, 193566210, 193566810, 193539610, 193540410, 193541210, 193565710, 193566310, 193566910, 193577510, 193577710, 193577910, 193583810, 193584010, 193584210, 190224250, 190227950, 190233710, 190235410, 193539810, 193540610, 193541410, 193565810, 193566410, 193567010, 193539910, 193540710, 193541510, 193565910, 193566510, 193567110, 193540010, 193540810, 193541610, 193566010, 193566610, 193567210, 193577610, 193577810, 193578010, 193583910, 193584110, 193584310, 190224350, 190228050, 190233810, 190235510, 190224410, 190224510,
COVID-19-related hospitalization charges	190232710, 190237610, 190237710,
COVID-19-related management charges for Intensive care unit	190223810, 190223910, 190224010, 190224110, 193532310, 193534710, 193537110, 193558510, 193560910, 193563310, 193532410, 193534810, 193537210, 193558610, 193561010, 193563410, 193532110, 193534510, 193536910, 193558310, 193560710, 193563110, 193532210, 193534610, 193537010, 193558410, 193560810, 193563210, 190223050, 190227150, 190223150, 190227250, 190233310, 190235010, 193575710, 193576310, 193576910, 193582010, 193582610, 193583210, 190223250, 190227350, 190223350, 190227450, 190233410, 190235110, 193532710, 193535110, 193537910, 193558910, 193561310, 193563710, 193532810, 193535210, 193537610, 193559010, 193561410, 193563810, 193532510, 193534910, 193537310, 193558710, 193561110, 193563510, 193532610, 193535010, 193537410, 193558810, 193561210, 193563610, 193575810, 193576410, 193577010, 193582110, 193582710, 193583310, 193533110, 193535510, 193537510, 193559310, 193561710, 193564110, 193533210, 193535610, 193538010, 193559410, 193561810, 193564210, 190230310, 190230510, 193532910, 193535310, 193537710, 193559110, 193561510, 193563910, 193533010, 193535410, 193537810, 193559210, 193561610, 193564010, 190230410, 190230610, 190236710, 190236810, 193575910, 193576510, 193577110, 193582210, 193582810, 193583410, 193533510, 193535910, 193538310, 193559710, 193562110, 193564510, 193533610, 193536010, 193538410, 193559810, 193562210, 193564610, 193533310, 193535710, 193538110, 193559510, 193561910, 193564310, 193533410, 193535810, 193538210, 193559610, 193562010, 193564410, 190223450, 190227550, 190223550, 190227650, 190233510, 190235210, 193576010, 193576610, 193577210, 193582310, 193582910, 193583510, 190223650, 190227750, 190223750, 190227850, 190233610, 190235310, 193533910, 193536310, 193538710, 193560110, 193562510, 193564910, 193534010, 193536410, 193538810, 193560210, 193562610, 193565010, 193533710, 193536110, 193538510, 193559910, 193562310, 193564710, 193533810, 193536210, 193538610, 193560010, 193562410, 193564810, 193576110, 193576710, 193577310, 193582410, 193583010, 193583610, 193534310, 193536710, 193539110, 193560510, 193562910, 193565310, 193534410, 193536810, 193539210, 193560610, 193563010, 193565410, 190230710, 190230910, 193534110, 193536510, 193538910, 193560310, 193562710, 193565110, 193534210, 193536610, 193539010, 193560410, 193562810, 193565210, 190230810, 190231010, 190236910, 190237010, 193576210, 193576810, 193577410, 193582510, 193583110, 193583710,
COVID-19-related management charges for maternal-fetal intensive care unit	190225610, 190225550, 190228750, 190234410, 190236110, 190225450, 190228650, 190234310, 190236010, 193545610, 193546210, 193546810, 193571210, 193571810, 193572410, 193545710, 193546310, 193546910, 193571310, 193571910, 193572510, 193545810, 193546410, 193547010, 193571410, 193572010, 193572610, 193579310, 193579510, 193579710,

	193585610, 193585810, 193586010, 193545910, 193546510, 193547110, 193571510, 193572110, 193572710, 193546010, 193546610, 193547210, 193571610, 193572210, 193572810, 193546110, 193546710, 193547310, 193571710, 193572310, 193572910, 193579410, 193579610, 193579810, 193585710, 193585910, 193586110,
COVID-19-related management charges for Neonatal intensive care unit	190225310, 193543910, 193544410, 193545110, 193569510, 193570010, 193570710, 193543810, 193544510, 193545010, 193569410, 193570110, 193570610, 193544010, 193544610, 193545210, 193569610, 193570210, 193570810, 193578710, 193578910, 193579110, 193585010, 193585210, 193585410, 190225150, 190228450, 190234110, 190235810, 193544110, 193544710, 193545310, 193569710, 193570310, 193570910, 193544210, 193544810, 193545410, 193569810, 193570410, 193571010, 193544310, 193544910, 193545510, 193569910, 193570510, 193571110, 193578810, 193579010, 193579210, 193585110, 193585310, 193585510, 190225250, 190228550, 190234210, 190235910,
COVID-19-related management charges for Pediatric intensive care unit	190225010, 193542810, 193543210, 193543610, 193568410, 193568810, 193569210, 193542910, 193543310, 193543710, 193568510, 193568910, 193569310, 193542610, 193543010, 193543410, 193568210, 193568610, 193569010, 193542710, 193543510, 193568310, 193569110, 193543110, 193568710, 190224850, 190228250, 190224950, 190228350, 190234010, 190235710, 193578410, 193578510, 193578610, 193584710, 193584810, 193584910,
COVID-19-related management charges for stroke care unit	190224710, 193541710, 193542010, 193542310, 193567310, 193567610, 193567910, 193541810, 193542110, 193542410, 193567410, 193567710, 193568010, 193541910, 193542210, 193542510, 193567510, 193567810, 193568110, 193578110, 193578210, 193578310, 193584410, 193584510, 193584610, 190224650, 190228150, 190233910, 190235610,
Treatment	
NIMV, supplemental oxygen	140005610, 140005810, 140057410, 140057310, 140005910, 140006050, 140037810, 140010050, 140024250, 140039550, 140039650
IMV, ECMO	140009010, 140010150, 140024350, 140009950, 140024150, 140009310, 140023510, 140009750, 140023950, 140009550, 140023750, 140039850, 140039950, 150147910, 150147610, 150147910, 150147610

COVID-19, coronavirus disease 2019; ECMO, extracorporeal membrane oxygenation; IMV, invasive mechanical ventilation; NIMV, non-invasive mechanical ventilation.

	Specificity (95% CI)	Numerator	Denominator	NPV (95% CI)	Numerator	Denominator
**Overall**						
Algorithm1	88.1 (87.9–88.3)	65,658	74,519	99.9 (99.9–99.9)	65,658	65,720
Algorithm2	99.4 (99.4–99.5)	74,098	74,519	99.9 (99.8–99.9)	74,098	74,190
Algorithm3	100.0 (99.9–100.0)	74,488	74,519	99.1 (99.1–99.2)	74,488	75,128
Algorithm4	99.4 (99.3–99.5)	74,071	74,519	99.9 (99.9–99.9)	74,071	74,152
**2020/1/1–2020/6/30**						
Algorithm1	88.1 (87.9–88.3)	65,658	74,519	99.9 (99.9–99.9)	65,658	65,720
Algorithm2	99.4 (99.4–99.5)	74,098	74,519	99.9 (99.8–99.9)	74,098	74,190
Algorithm3	100.0 (99.9–100.0)	74,488	74,519	99.1 (99.1–99.2)	74,488	75,128
Algorithm4	99.4 (99.3–99.5)	74,071	74,519	99.9 (99.9–99.9)	74,071	74,152
**2020/7/1–2020/12/31**						
Algorithm1	88.1 (87.9–88.3)	65,658	74,519	99.9 (99.9–99.9)	65,658	65,720
Algorithm2	99.4 (99.4–99.5)	74,098	74,519	99.9 (99.8–99.9)	74,098	74,190
Algorithm3	100.0 (99.9–100.0)	74,488	74,519	99.1 (99.1–99.2)	74,488	75,128
Algorithm4	99.4 (99.3–99.5)	74,071	74,519	99.9 (99.9–99.9)	74,071	74,152
**2021/1/1–2021/6/30**						
Algorithm1	88.1 (87.9–88.3)	65,658	74,519	99.9 (99.9–99.9)	65,658	65,720
Algorithm2	99.4 (99.4–99.5)	74,098	74,519	99.9 (99.8–99.9)	74,098	74,190
Algorithm3	100.0 (99.9–100.0)	74,488	74,519	99.1 (99.1–99.2)	74,488	75,128
Algorithm4	99.4 (99.3–99.5)	74,071	74,519	99.9 (99.9–99.9)	74,071	74,152
**2021/7/1–2021/12/31**						
Algorithm1	88.1 (87.9–88.3)	65,658	74,519	99.9 (99.9–99.9)	65,658	65,720
Algorithm2	99.4 (99.4–99.5)	74,098	74,519	99.9 (99.8–99.9)	74,098	74,190
Algorithm3	100.0 (99.9–100.0)	74,488	74,519	99.1 (99.1–99.2)	74,488	75,128
Algorithm4	99.4 (99.3–99.5)	74,071	74,519	99.9 (99.9–99.9)	74,071	74,152

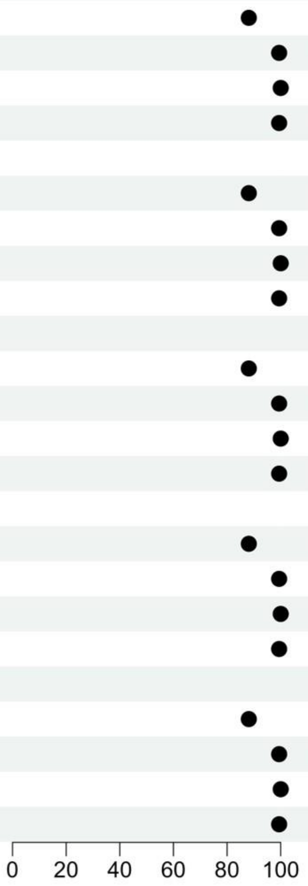

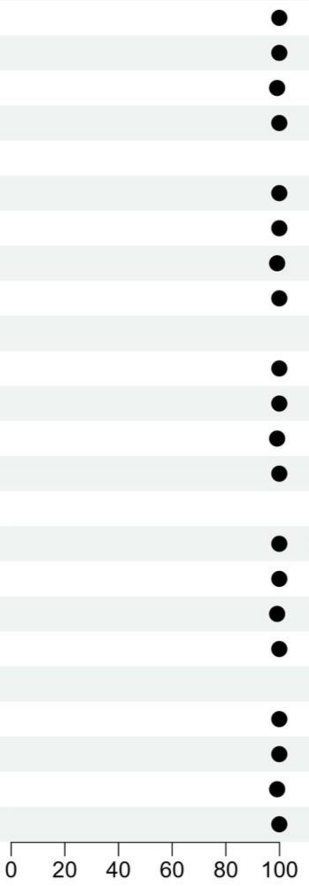

eFigure 1. Specificities and NPV of each algorithm for hospitalization with COVID-19. CI, confidence interval; COVID-19, coronavirus disease 2019; NPV, negative predictive value.

	Specificity (95% CI)	Numerator	Denominator	NPV (95% CI)	Numerator	Denominator
**Moderate or higher**						
**Overall**						
Algorithm3M	100.0 (99.9–100.0)	75,054	75,089	99.8 (99.8–99.9)	75,054	75,179
Algorithm4M	99.7 (99.6–99.7)	74,853	75,089	99.9 (99.9–99.9)	74,853	74,942
**2020/1/1–2020/6/30**						
Algorithm3M	100.0 (99.9–100.0)	74,597	74,626	99.8 (99.8–99.9)	75,011	75,128
Algorithm4M	99.7 (99.7–99.7)	74,400	74,626	99.9 (99.9–100.0)	74,342	74,383
**2020/7/1–2020/12/31**						
Algorithm3M	100.0 (99.9–100.0)	74,659	74,688	99.8 (99.8–99.9)	75,011	75,129
Algorithm4M	99.7 (99.7–99.7)	74,464	74,688	99.9 (99.9–99.9)	74,355	74,410
**2021/1/1–2021/6/30**						
Algorithm3M	100.0 (99.9–100.0)	74,700	74,730	99.8 (99.8–99.9)	75,013	75,132
Algorithm4M	99.7 (99.7–99.7)	74,506	74,730	99.9 (99.9–99.9)	74,313	74,374
**2021/7/1–2021/12/31**						
Algorithm3M	100.0 (99.9–100.0)	74,571	74,602	99.8 (99.8–99.9)	75,052	75,174
Algorithm4M	99.7 (99.7–99.7)	74,377	74,602	99.9 (99.9–100.0)	74,188	74,231
**Severe**						
**Overall**						
Algorithm3S	99.9 (99.9–100.0)	75,612	75,656	100 (100–100)	75,612	75,620
Algorithm4S	99.9 (99.9–99.9)	75,600	75,656	100 (100–100)	75,600	75,604
**2020/1/1–2020/6/30**						
Algorithm3S	100.0 (100.0–100)	74,658	74,661	100 (100–100)	75,136	75,143
Algorithm4S	100.0 (100.0–100)	74,647	74,661	100 (100–100)	74,529	74,532
**2020/7/1–2020/12/31**						
Algorithm3S	100.0 (100.0–100)	74,785	74,789	100 (100–100)	75,186	75,193
Algorithm4S	100.0 (100.0–100)	74,773	74,789	100 (100–100)	74,549	74,551
**2021/1/1–2021/6/30**						
Algorithm3S	100.0 (100.0–100)	74,924	74,936	100 (100–100)	75,287	75,295
Algorithm4S	100.0 (100.0–100)	74,913	74,936	100 (100–100)	74,543	74,546
**2021/7/1–2021/12/31**						
Algorithm3S	100.0 (99.9–100)	74,793	74,827	100 (100–100)	75,366	75,373
Algorithm4S	99.9 (99.9–100)	74,782	74,827	100 (100–100)	74,429	74,431

0 20 40 60 80 100 | 0 20 40 60 80 100

eFigure 2. Specificities and NPV for each algorithm for moderate or higher and severe status during hospitalization. "Moderate or higher" status includes "Severe" status. CI, confidence interval; NPV, negative predictive value.
